# Bridging the nutritional care gap: nurse-led education for potassium control in hemodialysis patients

**DOI:** 10.3389/fnut.2026.1752713

**Published:** 2026-03-10

**Authors:** Marouane Ouirdani, Amal Boutib, Fatima Ezzahra Fernane, Abderraouf Hilali, El Madani Saad, Abdelghafour Marfak, Ibtissam Youlyouz-Marfak

**Affiliations:** 1 Laboratory of Health Sciences and Technologies, Higher Institute of Health Sciences, Hassan First University of Settat, Settat, Morocco; 2 Euro-Mediterranean University of Fès (UEMF), Fez, Morocco; 3 National School of Public Health, Ministry of Health and Social Protection, Rabat, Morocco

**Keywords:** nurse-led education, hemodialysis, interdialytic weight gain, quality of life, serum potassium

## Abstract

**Background:**

In Morocco, a persistent gap exists between recommended standards of care and routine clinical practice in hemodialysis units, largely due to the high clinical workload of nephrologists and the absence of dietitians in several public hemodialysis centers. This shortage restricts individualized dietary and lifestyle counseling. This study aimed to explore the feasibility and potential clinical relevance of a simple, nurse-led educational intervention designed for hemodialysis centers operating without nutrition specialists.

**Methods:**

A quasi-experimental, single-arm, pre–post pilot study was conducted from February to June 2025 among 30 hemodialysis patients from three Moroccan centers with no permanent or visiting dietitians. The educational program focused on dietary potassium management, fluid intake control, thirst management strategies, and basic physical activity recommendations. Outcomes included serum potassium levels, quality of life (EQ-5D-5L), and interdialytic weight gain, assessed at baseline (T0) and post–intervention (T1).

**Results:**

Serum potassium levels significantly decreased after the intervention (*p* = 0.002), and the proportion of patients within the recommended range (4–5 mmol/L) increased from 36.7% at T0 to 46.7% at T1. No statistically significant changes were observed in quality of life or interdialytic weight gain.

**Conclusion:**

In this pilot study, a nurse-led educational intervention was associated with improved serum potassium control among hemodialysis patients in resource-limited settings lacking specialized nutrition personnel. However, no meaningful changes were observed in interdialytic weight gain or quality of life, suggesting that more intensive or individualized interventions may be required to influence these outcomes.

## 1 Introduction

In Morocco, statistical data on the causes of death among hemodialysis patients remains limited. However, circulatory diseases constitute the leading known cause of death, representing 17% of mortality in the general population, according to the Ministry of Health and Social Protection’s Health in Figures report (2023). It is well documented that individuals with chronic kidney disease (CKD) face a significantly higher risk of mortality from cardiovascular disease (CVD) compared to the general population (1). This elevated risk is largely attributable to the altered cardiovascular profile associated with CKD, where impaired renal function disrupts key homeostatic mechanisms. Notably, more than 50% of dialysis patients present with cardiovascular disease, and the relative risk of CVD-related mortality among hemodialysis (HD) patients is estimated to be approximately 20 times greater than that observed in the general population (2). Additionally, the cardiovascular status in CKD patients is multifactorial, involving key contributors such as hypertension, malnutrition, dyslipidemia, chronic kidney disease-mineral bone disorder (CKD-MBD), sedentary lifestyle, and insufficient physical activity (3).

Among mineral and bone disorders frequently observed in chronic kidney disease (CKD) (4), disturbances in serum potassium levels are particularly notable. Both hypokalemia (below 3.5 mmol/L) and hyperkalemia (greater than 5.0 mmol/L) negatively affect the cardiovascular system (5–7). Specifically, patients with end-stage renal disease (ESRD) undergoing maintenance hemodialysis are at significant risk of developing hyperkalemia, generally defined as a serum potassium concentration greater than 5.0 mmol/L (8). While definitions often rely on general population standards, clinical guidelines suggest that the optimal pre-dialysis serum potassium level should be maintained between 4.0 and 6.0 mmol/L (UK Renal Association), with stable normokalemia characterized by values in the 4.0–5.0 mmol/L range (9).

Clinical practice aims to identify blood potassium values linked to an elevated risk of death, as a U-shaped relationship exists between predialysis serum potassium levels and mortality, where both low and high levels are associated with higher risk (10). Studies suggest that the highest survival rate is linked to serum potassium levels between 4.6 and 5.3 mmol/L (11). Mortality risk becomes statistically significant when concentrations are <4.0 mmol/L or ≥5.6 mmol/L (11), and above 5.7 mmol/L (10, 12).

In addition to the fluctuations in serum potassium, interdialytic weight gain (IDWG) is also a vital therapeutic factor. Increased fluid retention between hemodialysis sessions correlates with a heightened risk of cardiovascular and overall mortality (13). Furthermore, a high IDWG is a substantial independent predictor of major adverse cardiac and cerebrovascular events (MACCE) (14). Consequently, managing IDWG is crucial for preventing cardiovascular complications and monitoring fluid status in hemodialysis patients.

Individuals with chronic diseases tend to adopt fewer health-promoting behaviors compared to the general population (15). Addressing this gap requires healthcare professionals to focus on empowering individuals to adopt and sustain habits such as medication adherence, physical activity, and dietary compliance (16). Patient education, whether focused on nutrition (17) or exercise (18), has proven effective in enhancing the overall health of hemodialysis patients. Effective collaboration with renal dietitians is essential, as individualized nutritional counseling covering sodium, phosphorus, potassium, and protein intake is fundamental to managing Chronic Kidney Disease–Mineral and Bone Disorder (CKD–MBD) (19, 20). Unfortunately, according to the Health in Figures report 2023, Morocco faces a significant shortage of dietitians (only 224 in the public sector for 133 HD centers), resulting in a large discrepancy between recommended standards of care and actual practice. Given the heavy workload of the limited number of nephrologists (average 2.5 per center), they are unable to provide regular, individualized dietary follow-up, justifying the need for this study.

In this context, the present study aims to evaluate the feasibility of a simple, nurse-led educational program implemented in hemodialysis centers without dietitians. The intervention is designed to support the regulation of serum potassium levels, maintain IDWG within recommended limits, and improve patients’ quality of life.

## 2 Materials and methods

### 2.1 Study design and ethics

This was an intervention study involving an educational program with hemodialysis patients. The research was conducted in accordance with the ethical principles outlined in the Declaration of Helsinki, and received formal approval from the Ethics Committee of the Euro-Mediterranean University of Fes (UEMF) (Approval No: CERS/UEMF-2025/EC10/02).

### 2.2 Study setting and design

The study was conducted in three public hemodialysis centers in Morocco between February and June 2025. These centers were intentionally selected because they operate without permanent or visiting dietitians, reflecting a common challenge in human resource-limited settings.

The study was designed as a pilot and feasibility investigation using a quasi-experimental, single-arm, pre–post design. All eligible participants received the educational intervention, and outcomes were assessed before (T0) and after (T1) the intervention. The study was not designed to test definitive effectiveness or to support causal inference, but rather to explore feasibility and observe preliminary trends under real-world constraints.

### 2.3 Participants and eligibility criteria

A total of 42 patients undergoing chronic hemodialysis were initially screened. Participants were included if they were on hemodialysis for at least three (3) months to ensure clinical stability and adaptation to the standard regimen. The cohort consisted primarily of adults (aged more than 18 years); however, one 13-year-old minor was included under the active participation and informed consent of her caregiver (mother). Exclusion criteria at baseline included refusal to participate, severe cognitive or sensory impairment (e.g., severe hearing loss) that would impede educational comprehension, and the active use of potassium binders.

### 2.4 Patient flow and final analysis cohort

Following the initial screening, one participant was excluded for not meeting the inclusion criteria. During the study period, an additional 11 participants were excluded from the final statistical analysis resulting in a final analysis cohort of 30 participants at T1. Reasons for exclusion included clinical events such as death (*n* = 2), change of hemodialysis center or modification of dialysis session frequency (*n* = 7), and the initiation of pharmacological treatments with a known indirect potassium-lowering effect at the time of the post-intervention assessment (*n* = 2).

### 2.5 Intervention

The educational intervention was developed based on evidence-based recommendations for preventing cardiovascular complications in hemodialysis patients. Participants received instructional materials in the form of posters and illustrated sheets. During the instructional sessions, visual content was also presented using a Samsung A3 tablet. Food selection was based on data from the Moroccan food composition Table (2019) (21), and the Ciqual food composition table (22). The educational intervention also addressed the physiological role of potassium, symptoms of hypokalemia and hyperkalemia, and practical strategies to reduce potassium content in foods (23–25).

Regarding physical activity, the recommendations were based on the Life Options Rehabilitation Advisory Council guidelines, emphasizing simple stretching exercises to promote flexibility and prevent discomfort. Patients were instructed to stop exercising and seek immediate medical consultation if they experienced symptoms such as angina, palpitations, dizziness, weakness, severe or unusual fatigue, or excessive shortness of breath during or following physical activity (26, 27). Education on fluid restriction and salt intake management was guided by recommendations from the National Kidney Foundation (28, 29). All educational content was delivered in Moroccan dialect, either verbally or using visual materials.

Educational sessions were delivered using standardized educational content across all participating centers. In the Settat center, sessions were conducted face-to-face at least once per week. In the two other centers, due to geographical constraints, the intervention was delivered using a combination of face-to-face sessions and remote communication (WhatsApp messages or phone calls). In all cases, receipt and comprehension of the educational messages were systematically confirmed with patients or their caregivers. The intervention was delivered by a general nurse, who also served as the principal investigator of the study.

### 2.6 Data collection

Interdialytic weight gain (IDWG) was calculated as the monthly mean value during the month preceding the intervention (T0) and during the final month of the study (T1). Serum potassium levels were measured at baseline and post-intervention, following KDIGO 2024 recommendations for blood sampling procedures. The target potassium range was defined as 4.0–5.0 mmol/L, which corresponds to the interval associated with the lowest mortality risk. Quality of life was assessed at both time points using the validated Arabic version of the EQ-5D-5L instrument (Registration No. 66845) and the Moroccan EQ-5D-5L value set (30).

### 2.7 Statistical analysis

Statistical analyses were performed using IBM SPSS Statistics for Windows, Version 25.0, and R software for the computation of EQ-5D-5L index scores. Normality of continuous variables was assessed using the Shapiro–Wilk test and visual inspection of histograms and Q–Q plots. Based on distribution characteristics, a paired samples t-test was applied to normally distributed variables (serum potassium levels and EQ-5D-5L index scores), whereas the Wilcoxon signed-rank test was used for non-normally distributed variables (IDWG). All statistical tests were two-tailed with a significance level set at *p* < 0.05. Only participants with complete paired measurements at both time points (T0 and T1) were included in the analysis, consistent with the study design.

## 3 Results

The study included 30 hemodialysis patients, the majority of whom were elderly individuals (53.33% were 65 years or older). Demographic, clinical, and diagnostic characteristics of the participants are presented in Table 1. Forty percent of participants were married, while 30% were widowed.

**Table 1:** Demographic and clinical characteristics of hemodialysis patients (*n* = 30).

Variable	Category/modality	Percentage (%)
Age, years	< 40	13.33%
	40 to 64	33.33%
	65 and older	53.33%
Marital status	Married	40%
	Widowed	30%
	Single	20%
	Divorced	10%
Educational level	No formal education	70%
	Primary school	16.66%
	Secondary school	6.66%
	University level	6.66%
Place of residence	Rural	43.33%
	Urban	56.66%
Presence of chronic disease	With at least one chronic disease	70%
	Without associated chronic disease	30%
Associated chronic diseases (*n* = 21)	Hypertension (HTN)	90.5%
	Cardiovascular disease	28.6%
	Diabetes	14.3%
	Thyroid disorder	4.8%
	Hypercholesterolemia	4.8%
Underlying cause of dialysis initiation	Unknown (Idiopathic)	40%
	Hypertension (HTN)	26.66%
	Diabetes mellitus (DM)	10%
	Diabetes + Hypertension	3.33%
	Nephrolithiasis/hydronephrosis	6.66%
	Polycystic kidney disease	3.33%
	Systemic lupus erythematosus	3.33%
	Congenital malformation	3.33%
	Nephrectomy	3.33%
Duration of dialysis treatment	1 to 2 years	23.33%
	3 to 5 years	26.66%
	6 to 10 years	13.33%
	More than 10 years	36.66%
Number of dialysis sessions per week	2 sessions/week	40%
	3 sessions/week	60%

Educational attainment was low, with 70% of participants having no formal education. Urban residents represented 56.66% of the sample. Most participants (70%) had at least one chronic condition, most commonly hypertension (90.5%), followed by cardiovascular disease (28.6%) and diabetes (14.3%). The most frequently reported cause of dialysis initiation was idiopathic (40%), followed by hypertension (26.66%) and diabetes (10%). Most patients had been receiving hemodialysis for more than 3 years, with 36.66% undergoing treatment for over 10 years. In 60% of cases, hemodialysis was performed three times per week.

A paired samples t-test was used to compare serum potassium levels before (T0) and after (T1) the educational intervention among the 30 participants (Table 2). A statistically significant reduction in serum potassium levels was observed after the intervention (*p* = 0.002), with a mean decrease of 0.38 mmol/L. The proportion of patients within the recommended potassium range (4.0–5.0 mmol/L) increased from 36.7% at T0 to 46.7% at T1. Among participants with serum potassium above 5.0 mmol/L at both T0 and T1, a reduction was observed in 91.7% of cases (Table 3).

**Table 2:** Comparative analysis of primary outcomes (serum potassium, IDWG, and quality of life) before and after educational intervention (T0 vs. T1).

Variable	Statistical test used	Before intervention (T0)	After intervention (T1)	Mean difference (T0 − T1)	Test statistic (t or z)	*p*-value
Serum potassium (mmol/L)	Paired t-test	5.39 ± 0.79	5.01 ± 0.71	0.38 ± 0.63	t = 3.312 (df = 29)	0.002
Interdialytic weight gain (kg)	Wilcoxon signed rank	1.16 ± 0.73	1.20 ± 1.25	−0.04 ± 1.12	Z = −0.524	0.600
EQ-5D-5L index score	Paired t-test	0.37 ± 0.62	0.30 ± 0.56	0.06 ± 0.51	t = 0.676 (df = 29)	0.504

The results are expressed as Mean ± Standard Deviation (SD). IDWG = Interdialytic Weight Gain. Serum Potassium and EQ-5D-5L Index scores were analyzed using a Paired t-test. Interdialytic Weight Gain (IDWG) was analyzed using the Wilcoxon Signed-Rank Test; the Z-value and *p*-value are derived from the asymptotic approximation of the test. The level of statistical significance was set at *p* < 0.05.

**Table 3:** Efficacy of intervention on serum potassium levels at baseline (T0) and post-intervention (T1).

Clinical outcome	Time point (T)	Count (N)	Percentage (%)
Serum K + in target range (≥4.0 to ≤ 5.0 mmol/L) (Overall *N* = 30)	T0 (Baseline)	11	36.7
	T1 (Post-Intervention)	14	46.7
Hyperkalemic sub-group (> 5.0 mmol/L at T0 and T1; *N* = 12)	Decrease in Serum K + (T0 to T1)	11	91.7
	Increase in Serum K + (T0 to T1)	1	8.3

T0 represents baseline (pre-intervention) data; T1 represents post-intervention (3-month follow-up) data. The target range for serum K + is defined as 4.0 to 5.0 mmol/L.

**Figure 1:**
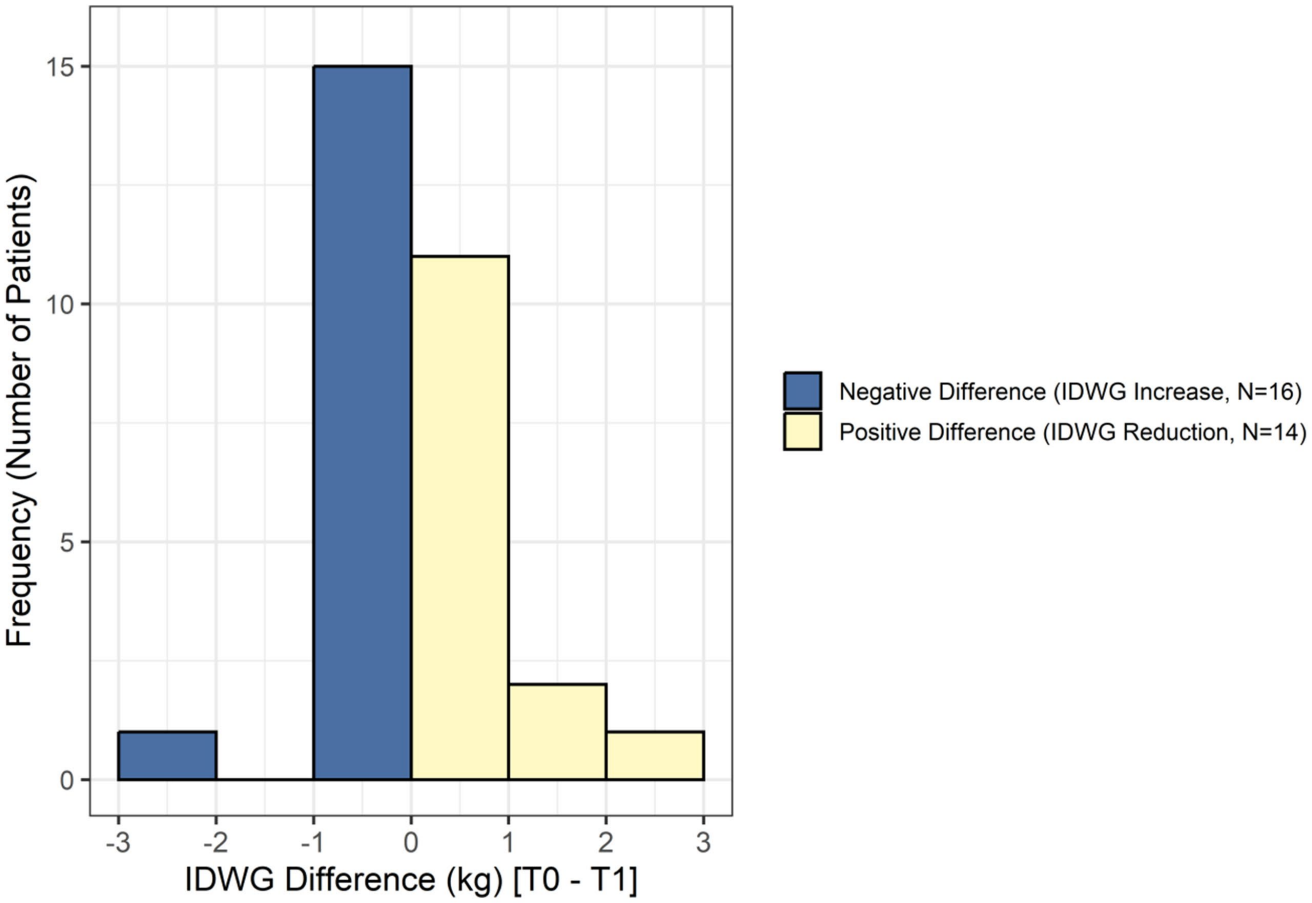
Related-samples Wilcoxon signed rank test (IDWG). Distribution of interdialytic weight gain differences (T0–T1) showing the number of patients with increased versus reduced IDWG following the nurse-led educational intervention.

Interdialytic weight gain (IDWG) was assessed using mean monthly values collected during the month preceding the intervention (T0) and during the final month of the intervention period (T1) (Table 2). As the normality assumption was not met, the Wilcoxon signed-rank test for paired samples was applied, using the difference T0 − T1, such that positive values indicated a reduction in IDWG. Fourteen participants (46.7%) showed a reduction in IDWG, while 16 participants (53.3%) showed an increase (Figure 1). No statistically significant difference in IDWG was observed between T0 and T1 (*p* = 0.6).

Quality of life, was assessed using the EQ-5D-5L index score. Paired t-test analysis showed a slight decrease in the mean EQ-5D-5L index score at T1 compared with T0; however, this change was not statistically significant (*p* = 0.504) (Table 2).

## 4 Discussion

In this pilot and feasibility study conducted under real-world public-sector constraints, an improvement in serum potassium levels was observed following a nurse-led educational intervention. Although causal inference cannot be established due to the single-arm pre–post design, the observed trend suggests potential clinical relevance in settings lacking access to specialized nutritional care. These observations are consistent with previous studies reporting similar trends following educational interventions aimed at controlling serum potassium levels. These investigations have reported either reduction in serum potassium levels (31–35) or a decrease in the number of patients with hyperkalemia (36). However, other research has suggested little or no association between dietary potassium intake and serum potassium in hemodialysis patients (37, 38).

In contrast to the observed improvement in serum potassium, no statistically significant changes were observed in quality of life (QoL) or interdialytic weight gain (IDWG). This finding differs from some studies reporting improvements in QoL following nutritional education interventions (34, 39, 40). Similarly, the absence of a significant change in IDWG contrasts with previous educational interventions (31, 41–45) and may partly be explained by contextual factors, including the absence of nephrologists in two centers during the first two months of the study, which may have affected dry-weight assessment. Low educational attainment among participants, with 70% having no formal education, may have limited comprehension and adherence to recommendations. In addition, the lack of improvement in QoL may be related to the high burden of comorbidities, and to the generally lower QoL reported among hemodialysis patients compared with transplant recipients and the healthy members of the general population (46, 47).

Overall, these findings suggest that a simplified nurse-led educational intervention is feasible in resource-limited hemodialysis settings and may be associated with improved potassium control. Nevertheless, this study has several limitations, including its limited sample size, potential contamination bias, socioeconomic constraints affecting adherence, variability in attendance at dialysis sessions, and the inability to assess psychosocial factors such as depressive status. In addition, differences in delivery modality across centers may have influenced patient engagement and could have introduced performance bias. Larger controlled studies with longer follow-up are needed to confirm effectiveness and sustainability of this approach.

## Data Availability

The datasets presented in this article are not readily available because the dataset contains sensitive clinical information from hemodialysis patients (serum potassium, interdialytic weight gain, and EQ-5D-5L scores). According to the requirements of the research ethics committee (CERS/UEMF-2025/EC10/02) and Moroccan regulations on the protection of personal health data, raw individual-level data cannot be shared publicly. Only aggregated and identified statistical summaries are available upon reasonable request, subject to ethical approval. Requests to access the datasets should be directed to m.ouirdani@uhp.ac.ma.
